# Expression profile of SIX family members correlates with clinic-pathological features and prognosis of breast cancer: A systematic review and meta-analysis

**DOI:** 10.1097/MD.0000000000004085

**Published:** 2016-07-08

**Authors:** Han-Xiao Xu, Kong-Ju Wu, Yi-Jun Tian, Qian Liu, Na Han, Xue-Lian He, Xun Yuan, Gen Sheng Wu, Kong-Ming Wu

**Affiliations:** a Department of Oncology, Tongji Hospital of Tongji Medical College, Huazhong University of Science and Technology, Wuhan, Hubei; b Nursing School of Pingdingshan University, Pingdingshan, Henan; c Clinical Research Center, Wuhan Medical and Healthcare Center for Women and Children, Wuhan, Hubei, People's Republic of China; d Departments of Oncology and Pathology, Karmanos Cancer Institute, Wayne State University School of Medicine, Detroit, MI.

**Keywords:** biomarker, breast cancer, molecular subtypes, prognosis, sineoculis homeobox homolog family members, tumor development

## Abstract

Sineoculis homeobox homolog (SIX) family proteins, including SIX1, SIX2, SIX3, SIX4, SIX5, and SIX6, have been implicated in the initiation and progression of breast cancer, but the role of each member in breast tumor is not fully understood. We conducted a systematic review and meta-analysis to evaluate the association between the mRNA levels of all 6 members and clinic-pathological characteristics and clinical outcome of breast cancer patients based on the PRISMA statement criteria.

ArrayExpress and Oncomine were searched for eligible databases published up to December 10, 2015. The association between the mRNA expression of SIX family members and clinic-pathological features and prognosis was measured by the odds ratio (OR), hazard ratio (HR), and the corresponding 95% confidence interval (CI), respectively. All statistical analyses were performed using STATA software.

In total, 20 published Gene Expression Omnibus (GEO) databases with 3555 patients were analyzed. Our analysis revealed that patients with *SIX1* overexpression had worse overall survival (OS) (HR: 1.28, 95% CI: 1.03–1.58) and shorter relapse-free survival (RFS) (HR: 1.28, 95% CI: 1.05–1.56), and much worse prognosis for luminal breast cancer patients with SIX1 overexpression (OS: HR: 1.64, 95% CI: 1.13–2.39; RFS: HR: 1.43, 95% CI: 1.06–1.93). We found that patients with higher *SIX2* level had shorter time to both relapse and metastasis. However, high *SIX3* mRNA level was a protective factor for OS and RFS of basal-like breast cancer patients.

Our study suggested that members of *SIX* family played distinct roles in breast cancer. Detailed analysis of the expression of the SIX family members might provide useful information to predict breast cancer progression and prognosis.

## Abstract 2

Supplemental Digital Content is available in the text

## 1 Introduction

Breast cancer is one of the most common neoplasms and the second leading cause of cancer-related mortality in women worldwide.^[1]^ Over the last several years, molecular signature proves the heterogeneity of breast cancer. Molecular classification provides better prediction of tumor behavior and is widely used to guide therapeutic strategies.^[2]^ However, the current identified molecular subtypes are still not sufficient to provide information in terms of application in cancer treatment. Therefore, identifying novel biomarkers that can predict the progression and prognosis of breast cancer is becoming increasingly urgent.^[3]^

Sineoculis homeobox homolog (SIX) family proteins are a group of evolutionarily conserved transcription factors that play important roles in cell proliferation, differentiation, apoptosis, adhesion, and migration. This family has 6 members, including SIX1, SIX2, SIX3, SIX4, SIX5, and SIX6.^[4]^ Each member plays a distinct role in the regulation of cell functions. For example, SIX1 is required for the development of murine kidney, muscle, and inner ear.^[5]^ Combinational activation of SIX1, SIX2, and SIX4 was confirmed to be essential to brain development^[6]^; absence or inactivation of these three genes partly accounted for various brain defects.^[6]^ It has been shown that loss of SIX3/6 expression can lead to pinhole-eye evolution in Nautilus.^[7]^

Aberrant expression of SIX class has been linked to cancer formation and progression.^[8,9]^ SIX1, the most studied SIX family member, was reported to play a role in the development of tumors, including pancreatic cancer,^[10]^ colorectal cancer,^[11]^ gastric cancer,^[12]^ and especially breast cancer.^[13–16]^ It promoted cell proliferation via reactivating the cell cycle-related proteins cyclin A^[17]^ and cyclin D,^[10]^ and stimulated malignant transformation of nontumorigenic cells.^[18]^ Ectopic expression of SIX1 led to tumor invasion and metastasis partly by modulating epithelial–mesenchymal transition (EMT).^[19,20]^ In addition, high SIX1 level is associated with paclitaxel resistance in breast cancer cells.^[15]^ More importantly, it was found to be closely linked to poor clinical prognosis of cancer patients.^[14,21]^ In patients with Wilms tumors, mutations of SIX1 and SIX2 may contribute to a higher rate of relapse and death.^[22]^ Further, SIX2 promoted breast cancer metastasis by downregulation of E-cadherin.^[23]^ However, high expression of SIX3 contributed to the improved clinical outcome of lung adenocarcinoma patients, and restoration of SIX3 in lung cancer cells led to the suppression of cell proliferation and migration.^[24]^ High protein abundance of SIX4 was closely correlated with poor differentiation and increased depth of invasion in esophageal squamous cell carcinoma.^[25]^

Although a variety of studies have been conducted to explore the association between SIX and breast cancer, the SIX family member expression signatures in breast cancer and their relation to molecular features remain unclear. Therefore, we conducted a meta-analysis to assess mRNA expression profile of *SIX* family in breast cancer and analyzed their correlation with molecular subtypes and clinical significance.

## 2 Methods

Ethical committee or institutional review board approvals were not necessary for this study because it was a meta-analysis based on existing literature.

### 2.1 Search strategy

The electronic databases including ArrayExpress and Oncomine were searched for relevant Gene Expression Omnibus (GEO) datasets of human breast cancer with the mRNA expression of *SIX* family members up to December 10, 2015, by using the search term “breast cancer.” Only the datasets which met the inclusion criteria were included in this meta-analysis.

### 2.2 Inclusion criteria

Databases we used fulfilled the following inclusion criteria: samples in the datasets were human breast cancer tissues or normal breast tissues; the mRNA expression of *SIX* family members was measured in these databases; the datasets were about mRNA, rather than DNA or microRNA; the sample capacity was more than 45; required clinic-pathological and prognosis information of breast cancer patients was available in these databases, such as grade, T stage, N stage, TNM stage, molecular subtypes, and clinical outcome. We only chose the most complete datasets, when several datasets had some patient population in common.

### 2.3 Data extraction

Data analysis was performed independently by 2 individuals. All data were extracted in a predefined table by using a standardized data collection form: first author's name, publication year, follow-up duration, tumor stage, patient number, detection methods, and platform. Cutoff values for *SIX1–6* were median expression. We reviewed ArrayExpress and Oncomine, and found 20 human breast cancer microarray datasets with mRNA expression of *SIX* family members and clinical data. For genes with more than 1 probe, the probe with maximum expression value was selected in our analysis. Overall survival (OS), relapse-free survival (RFS), and metastasis-free survival (MFS) were evaluated by Cox proportional hazard ratio (HR) and 95% confidence interval (CI).

The Newcastle-Ottawa Quality Assessment Scale (NOS) was employed to assess the quality of the studies. Based on the criteria, 8 sources of potential study bias estimating patient selection, study comparability, and outcomes were required to be identified.

### 2.4 Statistical analysis

The method we used to perform the statistical analysis was as described in our previous meta-analysis on CD44.^[26]^ The association between *SIX* mRNA expression and clinic-pathological parameters of breast cancer was assessed by the odds ratio (OR) and its corresponding 95% CI. HR was utilized to evaluate the effects of high expression of *SIX* family members on the clinical outcome of breast cancer patients and HR > 1 indicated that patients with higher mRNA expression of *SIX1–6* were more likely to have worse survival. Heterogeneity of publication across studies was assessed by a Chi-square-based-Q statistic and inconsistency index (*I*^2^) statistic. We employed the random-effect model if *I*^2^ value was more than 50% which indicated that heterogeneity could not be ignored. The fixed-effect model was considered when *I*^2^ value was less than 50% which suggested there was no heterogeneity or only moderate heterogeneity. Publication bias was measured by Begg test and Egger test. All statistical analyses were carried out using STATA software package (version 12.0) (Stata Corp LP, College Station, TX).

## 3 Results

### 3.1 Search result

**Figure 1:**
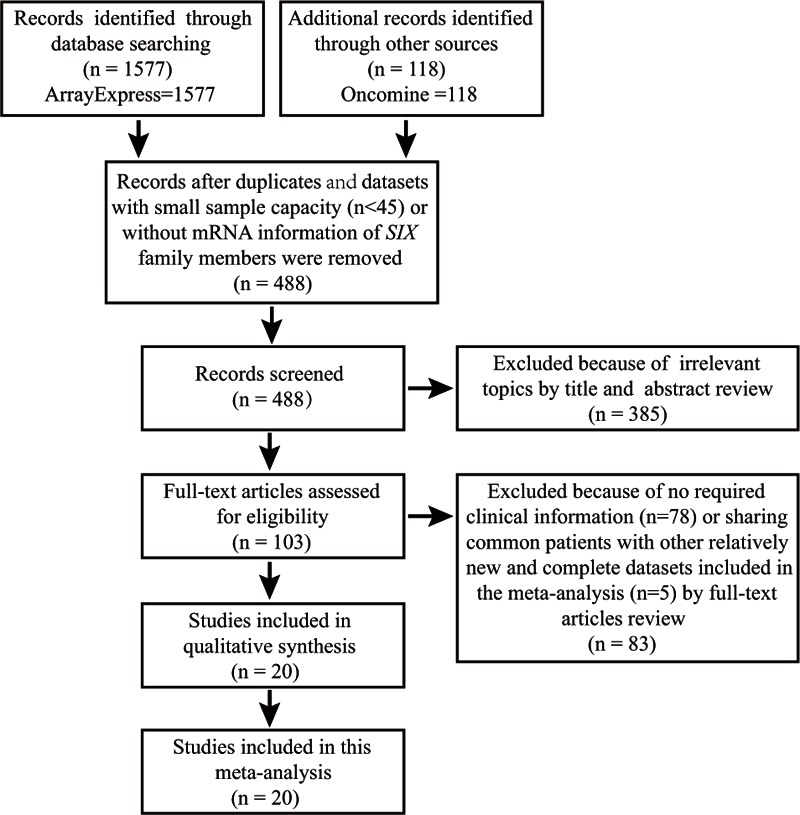
Flow diagram of article selection.

The flow diagram for the screening and identification of relevant studies is shown in Fig. 1. One thousand six hundred ninety-five datasets were initially identified, including 1577 records from ArrayExpress and 118 from Oncomine. A total of 1207 datasets were excluded because of duplicates, small sample capacity (n < 45) and data on DNA or microRNA level. We eliminated a total of 385 records after title and abstract screening because of irrelevant topics. After full-text review, a total of 83 datasets were excluded. Among these, 5 datasets were excluded because other datasets included in our meta-analysis contained the patient population from these 5 databases and we only chose the latest and most complete datasets, and other 78 databases were excluded due to no required clinical information. After the complicated screening, 20 studies with 3555 patients met the standard. Table 1 shows the characteristics of all 20 studies.^[27–46]^ These studies mainly assessed the association between the mRNA expression of *SIX1*, *SIX2*, *SIX3*, *SIX4*, *SIX5*, and *SIX6* with clinical parameters of breast cancer. Tumor size (T stage) 1 and 2 were identified as early T stage, and 3 and 4 were identified as late T stage. No lymph node metastasis (N0) was identified to be N-negative stage, while N1, N2, and N3 were classified into N-positive group. Tumor-node-metastasis (TNM) stages I and II were grouped as early-staged disease whereas III and IV were grouped as late-staged disease. Histological grade I and II were pooled as low-grade disease, while grade III was identified as high-grade disease.

**Table 1:**
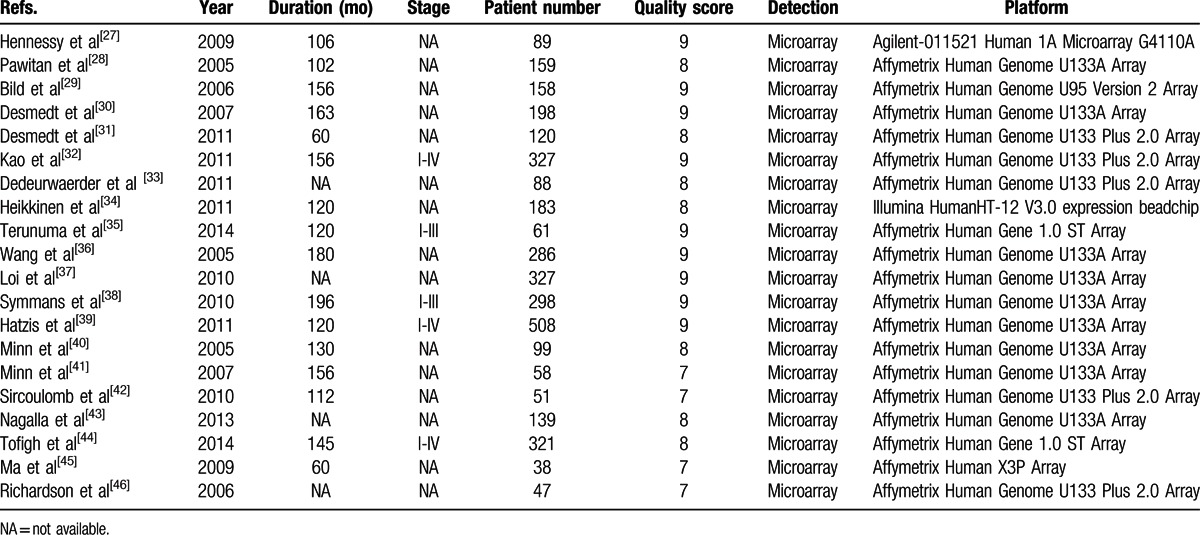
Characteristics of the included studies in the meta-analysis.

### 3.2 The mRNA levels of *SIX* family members are correlated with breast cancer risk

**Figure 2:**
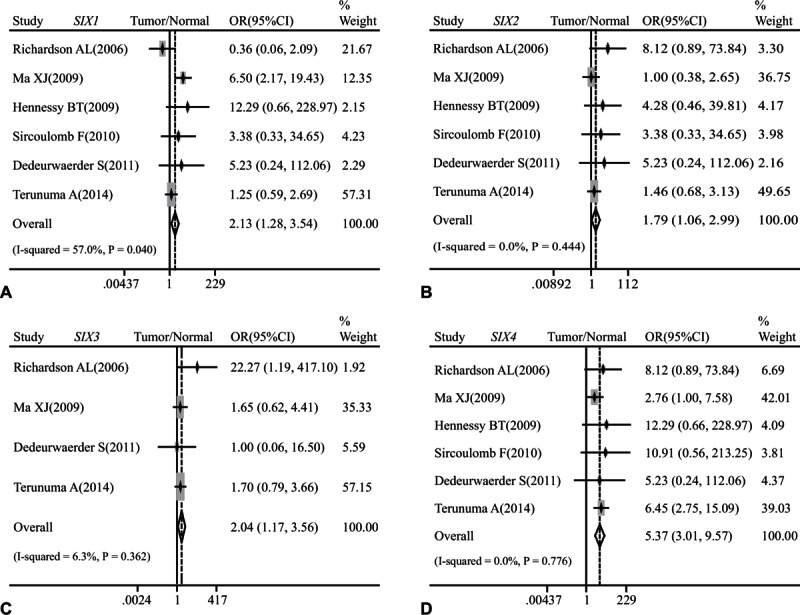
Forest plot of odds ratio (OR). CI = confidence interval. (A). Association between the mRNA expression of *SIX1* and breast cancer risks in comparison to normal breast tissues. (B). Association between the mRNA expression of *SIX2* and breast cancer risks in comparison to normal breast tissues. (C). Association between the mRNA expression of *SIX3* and breast cancer risks in comparison to normal breast tissues. (D). Association between the mRNA expression of *SIX4* and breast cancer risks in comparison to normal breast tissues.

There are a total of 6 studies that assessed the association between the mRNA level of *SIX* family members and breast cancer risk. Our analysis indicated that the mRNA expression of *SIX1* (OR: 2.13, 95% CI: 1.28–3.54; *P* = 0.040 and *I*^2^ = 57.0%; Fig. 2A), *SIX2* (OR: 1.79, 95% CI: 1.06–2.99; *P* = 0.444 and *I*^2^ = 0.0%; Fig. 2B), *SIX3* (OR: 2.04, 95% CI: 1.17–3.56; *P* = 0.362 and *I*^2^ = 6.3%; Fig. 2C), and *SIX4* (OR: 5.37, 95% CI: 3.01–9.57; *P* = 0.776 and *I*^2^ = 0.0%; Fig. 2D) was increased in breast cancer tissues when compared with normal breast tissues.

### 3.3 The mRNA levels of *SIX* family members are correlated with clinic-pathological features in breast cancer

**Figure 3:**
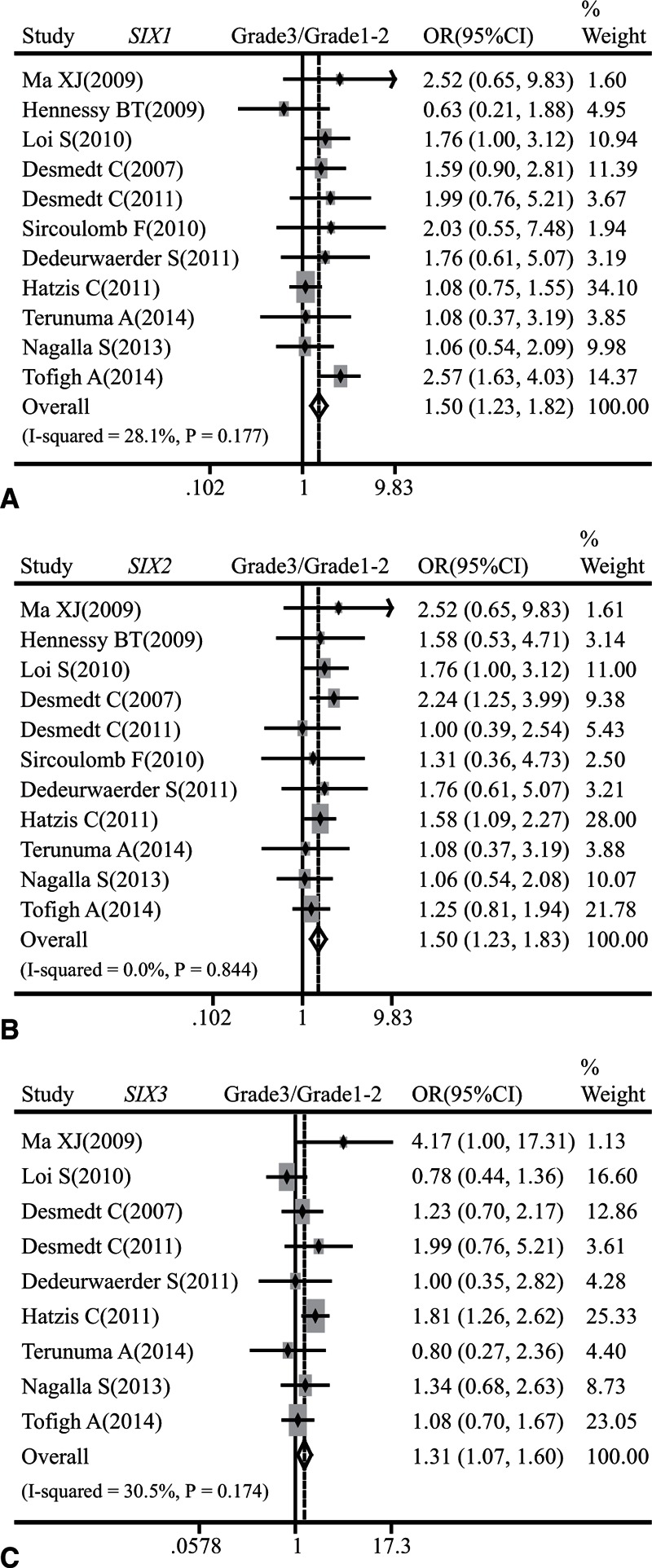
Forest plot of odds ratio (OR). CI = confidence interval. (A). Association between the mRNA expression of *SIX1* and histological grade of breast cancer. (B). Association between the mRNA expression of *SIX2* and histological grade of breast cancer. (C). Association between the mRNA expression of *SIX3* and histological grade of breast cancer.

Our results suggested that breast cancer patients with higher histological grade were likely to have a larger amount of *SIX1* (OR: 1.50, 95% CI: 1.23–1.82; *P* = 0.177 and *I*^2^ = 28.1%; Fig. 3A), *SIX2* (OR: 1.50, 95% CI: 1.23–1.83; *P* = 0.844 and *I*^2^ = 0.0%; Fig. 3B), or *SIX3* (OR: 1.31, 95% CI: 1.07–1.60; *P* = 0.174 and *I*^2^ = 30.5%; Fig. 3C) at mRNA level. But, we failed to find any association between the mRNA expression of *SIX1–6* and T stage (Supplementary Figure 1), N status (Supplementary Figure 2), or TNM stage (Supplementary Figure 3).

### 3.4 The mRNA expression of *SIX* family members is correlated with molecular subtypes of breast cancer

**Figure 4:**
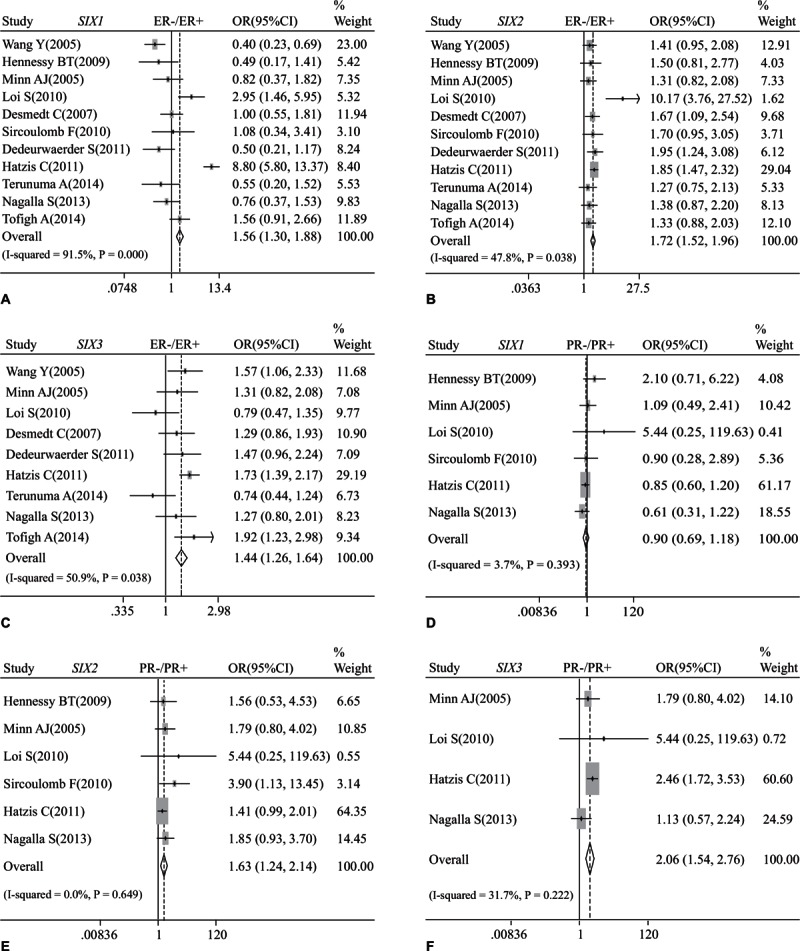
Forest plot of odds ratio (OR). CI = confidence interval. (A). Association between the mRNA expression of *SIX1* and ER status of breast cancer. (B). Association between the mRNA expression of *SIX2* and ER status of breast cancer. (C). Association between the mRNA expression of *SIX3* and ER status of breast cancer. (D). Association between the mRNA expression of *SIX1* and PR status of breast cancer. (E). Association between the mRNA expression of *SIX2* and PR status of breast cancer. (F). Association between the mRNA expression of *SIX3* and PR status of breast cancer.

The association between *SIX* mRNA expression with the status of estrogen receptor (ER), progesterone receptor (PR), human epidermal growth factor receptor-2 (HER2), and basal-like breast cancer was also analyzed. The mRNA levels of *SIX1* (OR: 1.56, 95% CI: 1.30–1.88; *P* < 0.001 and *I*^2^ = 91.5%; Fig. 4A), *SIX2* (OR: 1.72, 95% CI: 1.52–1.96; *P* = 0.038 and *I*^2^ = 47.8%; Fig. 4B), and *SIX3* (OR: 1.44, 95% CI: 1.26–1.64; *P* = 0.038 and *I*^2^ = 50.9%; Fig. 4C) were negatively correlated with the status of ER. As for PR status, the mRNA expression of *SIX2* (OR: 1.63, 95% CI: 1.24–2.14; *P* = 0.649 and *I*^2^ = 0.0%; Fig. 4E) and *SIX3* (OR: 2.06, 95% CI: 1.54–2.76; *P* = 0.222 and *I*^2^ = 31.7%; Fig. 4F) was inversely correlated with PR status. No significant association was found between PR status and *SIX1* (OR: 0.90, 95% CI: 0.69–1.18; *P* = 0.393 and *I*^2^ = 3.7%; Fig. 4D). Furthermore, the mRNA levels of *SIX1* (OR: 0.66, 95% CI: 0.48–0.92; *P* = 0.030 and *I*^2^ = 54.9%; Supplementary Figure 4A) and *SIX2* (OR: 0.61, 95% CI: 0.45–0.84; *P* = 0.196 and *I*^2^ = 29.1%; Supplementary Figure 4B) were positively correlated with HER2 status, but we failed to find significant association between HER2 status and the mRNA expression of *SIX3* (OR: 1.16, 95% CI: 0.84–1.61; *P* = 0.164 and *I*^2^ = 36.4%; Supplementary Figure 4C), *SIX4* (OR: 1.02, 95% CI: 0.93–1.12; *P* = 0.594 and *I*^2^ = 0.0%; Supplementary Figure 4D), *SIX5* (OR: 1.01, 95% CI: 0.96–1.06; *P* = 0.839 and *I*^2^ = 0.0%; Supplementary Figure 4E), and *SIX6* (OR: 1.01, 95% CI: 0.96–1.05; *P* = 0.787 and *I*^2^ = 0.0%; Supplementary Figure 4F).

**Figure 5:**
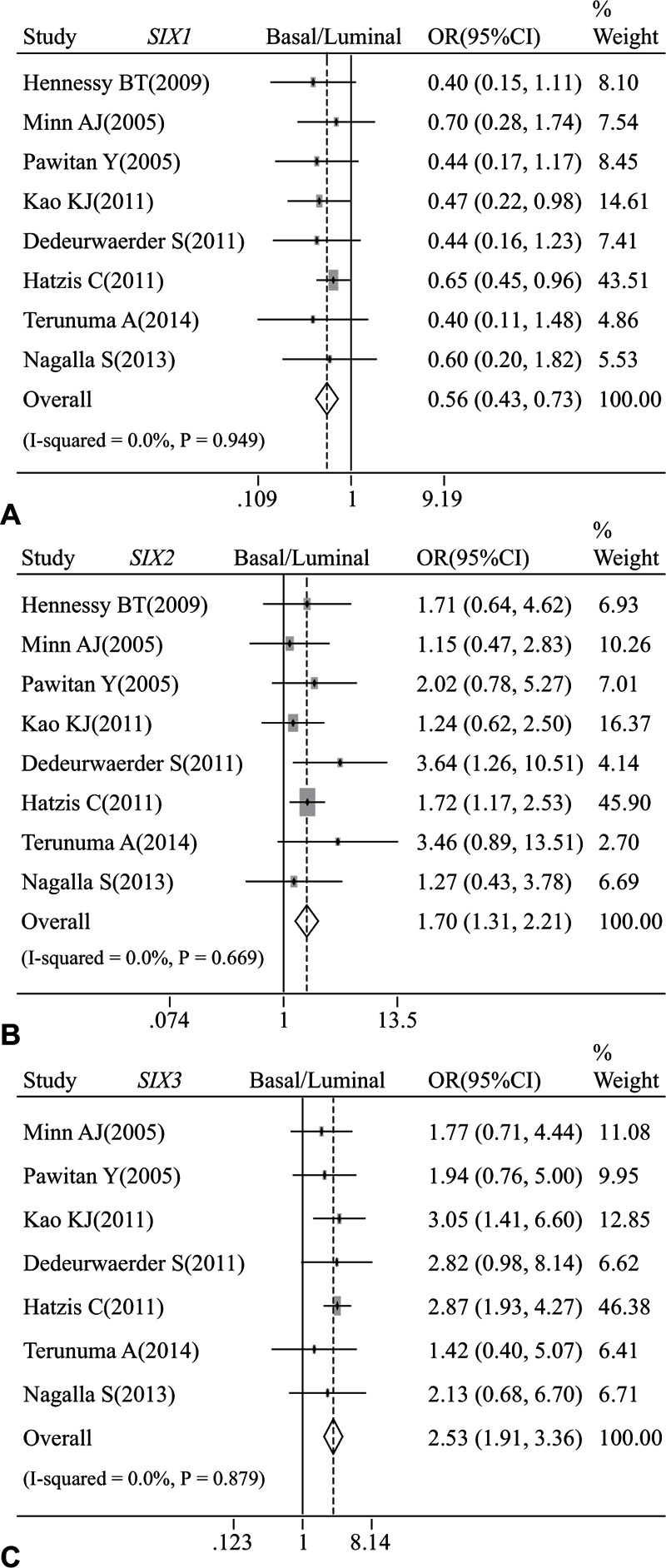
Forest plot of odds ratio (OR). CI = confidence interval. (A). Association between the mRNA expression of *SIX1* and basal-like breast cancer in comparison to luminal subtype. (B). Association between the mRNA expression of *SIX2* and basal-like breast cancer in comparison to luminal subtype. (C). Association between the mRNA expression of *SIX3* and basal-like breast cancer in comparison to luminal subtype.

Furthermore, the mRNA expression of *SIX2* (OR: 1.70, 95% CI: 1.31–2.21; *P* = 0.669 and *I*^2^ = 0.0%; Fig. 5B) and *SIX3* (OR: 2.53, 95% CI: 1.91–3.36; *P* = 0.879 and *I*^2^ = 0.0%; Fig. 5C) was statistically higher in basal-like tumors than in the luminal subtype of breast cancer, while that of *SIX1* (OR: 0.56, 95% CI: 0.43–0.73; *P* = 0.949 and *I*^2^ = 0.0%; Fig. 5A) was obviously lower in basal-like breast cancer in comparison with luminal subtype.

### 3.5 The mRNA expression of *SIX* family members is correlated with breast cancer survival

**Figure 6:**
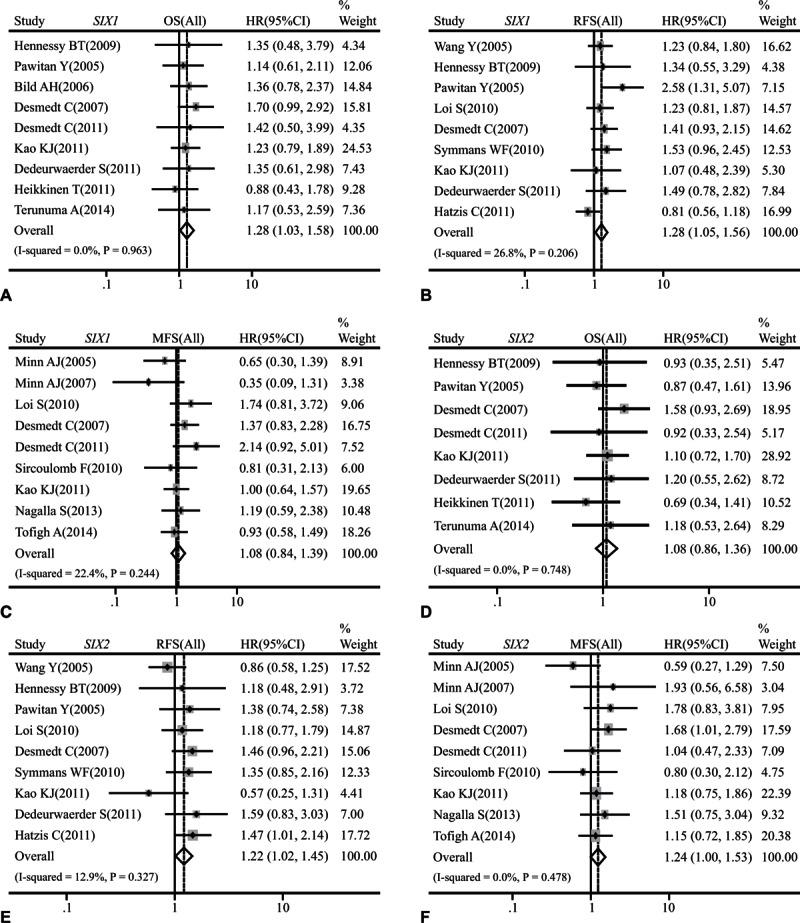
Forest plot of hazard radio (HR). CI = confidence interval. (A). Association between the mRNA expression of *SIX1* and OS of breast cancer. (B). Association between the mRNA expression of *SIX1* and RFS of breast cancer. (C). Association between the mRNA expression of *SIX1* and MFS of breast cancer. (D). Association between the mRNA expression of *SIX2* and OS of breast cancer. (E). Association between the mRNA expression of *SIX2* and RFS of breast cancer. (F). Association between the mRNA expression of *SIX2* and MFS of breast cancer.

Our analysis indicated that *SIX1*, *SIX2*, and *SIX4* were associated with clinical prognosis of whole breast cancer population at mRNA level. High mRNA level of *SIX1* was statistically associated with a poor OS (HR: 1.28, 95% CI: 1.03–1.58; *P* = 0.963 and *I*^2^ = 0.0%; Fig. 6A) and RFS (HR: 1.28, 95% CI: 1.05–1.56; *P* = 0.206 and *I*^2^ = 26.8%; Fig. 6B) of whole population of breast cancer. However, we could not find any significant association between *SIX1* mRNA expression and MFS of whole breast cancer population (HR: 1.08, 95% CI: 0.84–1.39; *P* = 0.244 and *I*^2^ = 22.4%; Fig. 6C). Furthermore, *SIX2* was statistically associated with RFS (HR: 1.22, 95% CI: 1.02–1.45; *P* = 0.327 and *I*^2^ = 12.9%; Fig. 6E) and MFS (HR: 1.24, 95% CI: 1.00–1.53; *P* = 0.478 and *I*^2^ = 0.0%; Fig. 6F), but not correlated with OS (HR: 1.08, 95% CI: 0.86–1.36; *P* = 0.748 and *I*^2^ = 0.0%; Fig. 6D) of whole breast cancer population. Furthermore, patients with higher *SIX4* level tended to display worse OS (HR: 1.39, 95% CI: 1.04–1.86; *P* = 0.770 and *I*^2^ = 0.0%; Supplementary Figure 5A) of whole breast cancer population, while did not exhibit significant difference on RFS (HR: 1.24, 95% CI: 0.80–1.92; *P* = 0.689 and *I*^2^ = 0.0%; Supplementary Figure 5B) and MFS (HR: 0.84, 95% CI: 0.59–1.20; *P* = 0.266 and *I*^2^ = 24.3%; Supplementary Figure 5C).

**Figure 7:**
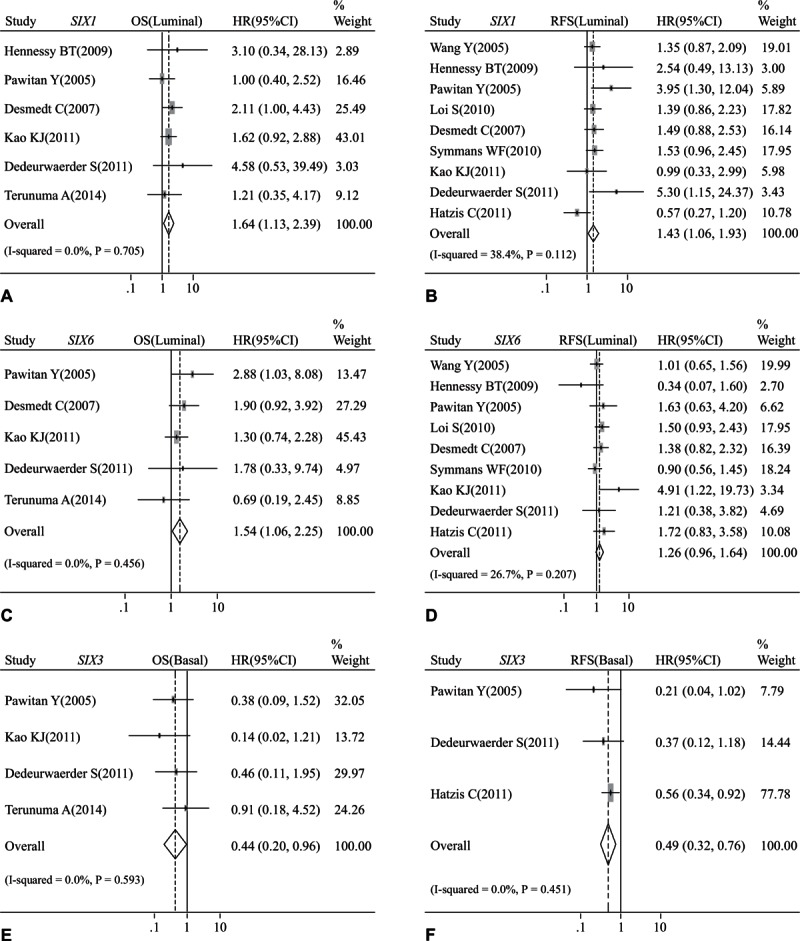
Forest plot of hazard radio (HR). CI = confidence interval. (A). Association between the mRNA expression of *SIX1* and OS of luminal breast cancer. (B). Association between the mRNA expression of *SIX1* and RFS of luminal breast cancer. (C). Association between the mRNA expression of *SIX6* and OS of luminal breast cancer. (D). Association between the mRNA expression of *SIX6* and RFS of luminal breast cancer. (E). Association between the mRNA expression of *SIX3* and OS of basal-like breast cancer. (F). Association between the mRNA expression of *SIX3* and RFS of basal-like breast cancer.

Moreover, subgroup analysis showed that some *SIX* class members had impact on survival performance of patients with a certain molecular subtype. High *SIX1* contributed to poor OS (HR: 1.64, 95% CI: 1.13–2.39; *P* = 0.705 and *I*^2^ = 0.0%; Fig. 7A) and RFS (HR: 1.43, 95% CI: 1.06–1.93; *P* = 0.112 and *I*^2^ = 38.4%; Fig. 7B) of luminal breast cancer patients. *SIX6* was also found to be linked to poor OS of patients with luminal breast cancer (HR: 1.54, 95% CI: 1.06–2.25; *P* = 0.456 and *I*^2^ = 0.0%; Fig. 7C), but not associated with RFS (HR: 1.26, 95% CI: 0.96–1.64; *P* = 0.207 and *I*^2^ = 26.7%; Fig. 7D) of this subgroup. On the contrary, high *SIX3* level was found to be associated with better OS (HR: 0.44, 95% CI: 0.20–0.96; *P* = 0.593 and *I*^2^ = 0.0%; Fig. 7E) and RFS (HR: 0.49, 95% CI: 0.32–0.76; *P* = 0.451 and *I*^2^ = 0.0%; Fig. 7F) of basal-like breast cancer patients.

### 3.6 Publication bias

Publication bias statistics were obtained using Begg test and Egger test. There is no significant publication bias for the following analysis: mRNA expression of *SIX* family members: breast cancer risk: SIX1: Begg test *P* = 0.707, Egger test *P* = 0.568; SIX3: Begg test *P* = 0.734, Egger test *P* = 0.474; SIX4: Begg test *P* = 0.707, Egger test *P* = 0.381. Histologic grade: SIX1: Begg test *P* = 1.000, Egger test *P* = 0.872; SIX2: Begg test *P* = 0.755, Egger test *P* = 0.894; SIX3: Begg test *P* = 0.754, Egger test *P* = 0.996. ER status: SIX1: Begg test *P* = 0.276, Egger test *P* = 0.058; SIX2: Begg test *P* = 0.755, Egger test *P* = 0.578; PR status: SIX3: Begg test *P* = 1.000, Egger test *P* = 0.789. Basal-like breast cancer: SIX2: Begg test *P* = 0.266, Egger test *P* = 0.549; SIX3: Begg test *P* = 0.133, Egger test *P* = 0.072. OS (All): SIX1: Begg test *P* = 0.754, Egger test *P* = 0.814. RFS (All): SIX1: Begg test *P* = 0.466, Egger test *P* = 0.231; SIX2: Begg test *P* = 0.466, Egger test *P* = 0.699. MFS (All): SIX2: Begg test *P* = 0.602, Egger test *P* = 0.756. OS (luminal): SIX1: Begg test *P* = 0.707, Egger test *P* = 0.523; SIX6: Begg test *P* = 1.000, Egger test *P* = 0.931. RFS (luminal): SIX1: Begg test *P* = 0.348, Egger test *P* = 0.362; OS (basal): SIX3: Begg test *P* = 1.000, Egger test *P* = 0.450. RFS (basal): SIX3: Begg test *P* = 0.296, Egger test *P* = 0.121.

## 4 Discussion

Members of the SIX family are expressed at the low level in normal adult tissues but increased in human cancers.^[47,48]^ We found that mRNA levels of *SIX1*, *SIX2*, *SIX3*, and *SIX4* were higher in breast cancer as compared to normal counterparts, suggesting their overexpression may contribute to the development of breast cancer. Consistent with this notion, Jin et al^[14]^ analyzed SIX1 expression by immunohistochemistry analysis in 262 breast cancer tissues and found that SIX1 protein was elevated in breast cancer. The mechanism by which SIX1 promoted breast tumor formation may be reinstating its properties normally displayed in early developmental tissues, including stimulation of proliferation and inhibition of apoptosis.^[49]^ SIX1 transcriptionally induces the expression of growth-promoting genes, such as cyclin A1, cyclin D1, and c-Myc.^[50,51]^ By increasing these gene expression, SIX1 promoted malignant transformation.^[17,18]^

Based on our results, histological grade of breast cancer tended to be positively associated with the mRNA expression of *SIX1–3*, which may indicate that high *SIX1–3* levels were linked to poor differentiation. In agreement, immunohistochemistry analysis on breast phyllodes cancer showed that tumor grade was positively correlated with SIX1 protein level.^[16]^ By activating proproliferative and prosurvival mechanisms, SIX family members promoted expansion of progenitor cell populations prior to differentiation.^[52–54]^ In addition to breast cancer, higher SIX1 level was also linked to poor differentiation in gastric tumor^[47]^ and prostate cancer.^[55]^

Currently, association between the *SIX* family members and ER status, PR status or basal-like breast cancer remains unclear. Based on our analysis, *SIX1*, *SIX2*, and *SIX3* were negatively linked to ER status at mRNA level. *SIX2* and *SIX3* were negatively correlated with PR status. ER+/PR+ breast tumors were most likely to be low grade.^[2]^ We also found that expressions of *SIX1–3* were positively correlated with histological grade and inversely correlated with the status of ER and PR. Based on the status of ER, PR, and HER2, breast cancers are grouped into 5 distinct molecular subtypes, namely luminal A, luminal B, HER2-overexpressing, basal-like, and normal-like.^[2]^ Among these subtypes, luminal breast cancer accounted for the majority of breast cancer and tended to be with a better outcome, while patients with basal-like subtype have a poor survival rate.^[2]^ In this study, we found that in contrast to high expression of *SIX2* and *SIX3*, the level of *SIX1* mRNA was significantly lower in basal-like tumors as compared to luminal subtype. However, the expression of *SIX1* mRNA was positively associated with HER2 status. A further study revealed that high level of SIX1 protein was significantly associated with HER2+ status.^[14]^ About 67.2% of HER2+ breast tissues were SIX1 strongly positive, while only 49.4% of HER2− tumor tissues were with strong staining of SIX1.^[14]^ We assumed that high *SIX1* mRNA level of HER2-overexpressing compensated the low *SIX1* mRNA of basal-like breast cancer, contributing to the negative correlation between *SIX1* mRNA and ER status at general level. Tumors of basal-like subtype are highly heterogeneous and tend to be high grade.^[2]^ Additionally, our results showed that elevated level of *SIX2* and *SIX3* was correlated with higher histological grade. Thus, it is not surprising that the mRNA levels of *SIX2* and *SIX3* was much higher in basal-like tumors than in luminal one.

Our results indicated that some *SIX* members had distinct impact on the survival of breast cancer patients. For example, high *SIX1* mRNA level was significantly correlated with poor OS and RFS of breast cancer population, but not correlated with MFS. This is consistent with a study on 262 breast cancer tissues showing that breast cancer patients with higher SIX1 protein level had remarkably lower 5-year OS rate than those with low SIX1 expression.^[14]^ Furthermore, patients with higher *SIX1* mRNA level were also found to exhibit obviously worse RFS. By activating transforming growth factor-beta (TGF-β) and mitogen-activated protein kinase (MEK)/ERK signaling, SIX1 obviously enriched breast cancer stem population.^[13]^ However, *SIX1* level did not have effects on MFS. Aberrant expression of SIX1 was found not only in about half of primary breast cancer, but also even in the majority of metastatic lesions.^[56]^ SIX1 was found to potently promote the metastatic spread of breast cancer MCF-7 cells.^[19]^ Several molecular studies on SIX1 could explain why *SIX1* has unfavorable impact on breast cancer patient metastasis. SIX1 suppressed the expression of epithelial marker E-cadherin by activating TGF-β, which promoted EMT and finally resulted in tumor metastasis.^[57]^ In addition, SIX1 promoted lymphanogenesis by upregulating vascular endothelial growth factor (VEGF)-C to contribute to tumor metastasis.^[57,58]^ However, tumor metastasis was regulated by a complex network. A large variety of molecules were involved in this process, such as epithelial growth factor receptor (EGFR) and TGF-β.^[59]^ Considering this complex regulation of breast cancer metastasis process, the effects of *SIX1* on MFS might be covered.

In addition, patients with high *SIX2* mRNA expression tended to have shorter time to both relapse and metastasis at overall level. SIX2 was reported to be a novel regulator of human breast tumor metastasis.^[23]^ SIX2 can promote tumor metastasis by downregulating the epithelial marker E-cadherin. The underlying mechanisms involve the upregulation of Zeb2 that is a direct suppressor of E-cadherin and direct promotion of the methylation of E-cadherin.^[23]^

Additionally, subcategory analysis indicated that some members play crucial roles in the survival performance of a certain molecular subtype group. For instance, *SIX1* was associated with poor OS and RFS of luminal breast cancer patients. *SIX6* was linked to poor OS of luminal cancer patients. *SIX1*'s unfavorable impact on clinical outcome of luminal group was supported by Iwanaga R’ research.^[13]^ Apart from these, higher *SIX3* mRNA level was strikingly found to contribute to a better OS and RFS in basal-like breast cancer population, indicating that *SIX3* is an anticancer factor for basal-like breast tumor. Although the protective role of *SIX3* in the clinical outcome of basal-like breast cancer has not been reported, this role in lung adenocarcinoma has been identified.^[24]^

Both heterogeneity tests and publication bias are essential to a meta-analysis. In this study, evidence of minor heterogeneities was noted. The production of heterogeneity in this result might be due to the following aspects: the platforms used to assess the *SIX* expression were different. Different platforms mean different design of probe sets for a certain gene; the sample size is limited, indicating that multicenter prospective studies are needed; the demographic data from different datasets were diverse, such as sex, age, disease stage; patients came from different countries. The expression level of a certain gene may be different in different races. In this meta-analysis, no big significant publication bias was found, suggesting our results may be very close to reality.

## 5 Conclusions

Taken together, our meta-analysis provides evidence that *SIX* family members play distinct and crucial roles in progression and prognosis of breast cancer. *SIX1*, *SIX2*, and *SIX4* are activated in breast cancer patients. Increased *SIX1–3* expression is linked to high histological grade and ER status, and that *SIX2* and *SIX3* are upregulated in basal-like breast cancer. High levels of *SIX1* and *SIX2* predict poor clinical outcome. *SIX1* and *SIX6* could serve as an unfavorable factor for prognosis of luminal breast cancer patients, while *SIX3* is capable of playing a protective role in prognosis of basal-like breast cancer patients. Our meta-analysis reveals an association between SIX family members and clinic-pathological features and prognosis. The role of SIX family as biomarkers for predicting breast cancer progression and prognosis is worthy of further validation.

## Supplementary Material

Supplemental Digital Content
