# Supplementary material for: Expression profile of SIX family members correlates with clinic-pathological features and prognosis of breast cancer: A systematic review and meta-analysis
Source: Medicine (Baltimore). 2016 Jul 8;95(27):e4085. doi: 10.1097/MD.0000000000004085 (PMC5058828; doi:10.1097/MD.0000000000004085)

**Supplementary Figure Legends**

**Supplementary Figure 1** Forest plots of the odds ratio (OR) and 95% confidence interval (CI) for the association between the mRNA levels of *SIX* family members and T stage of breast cancer.


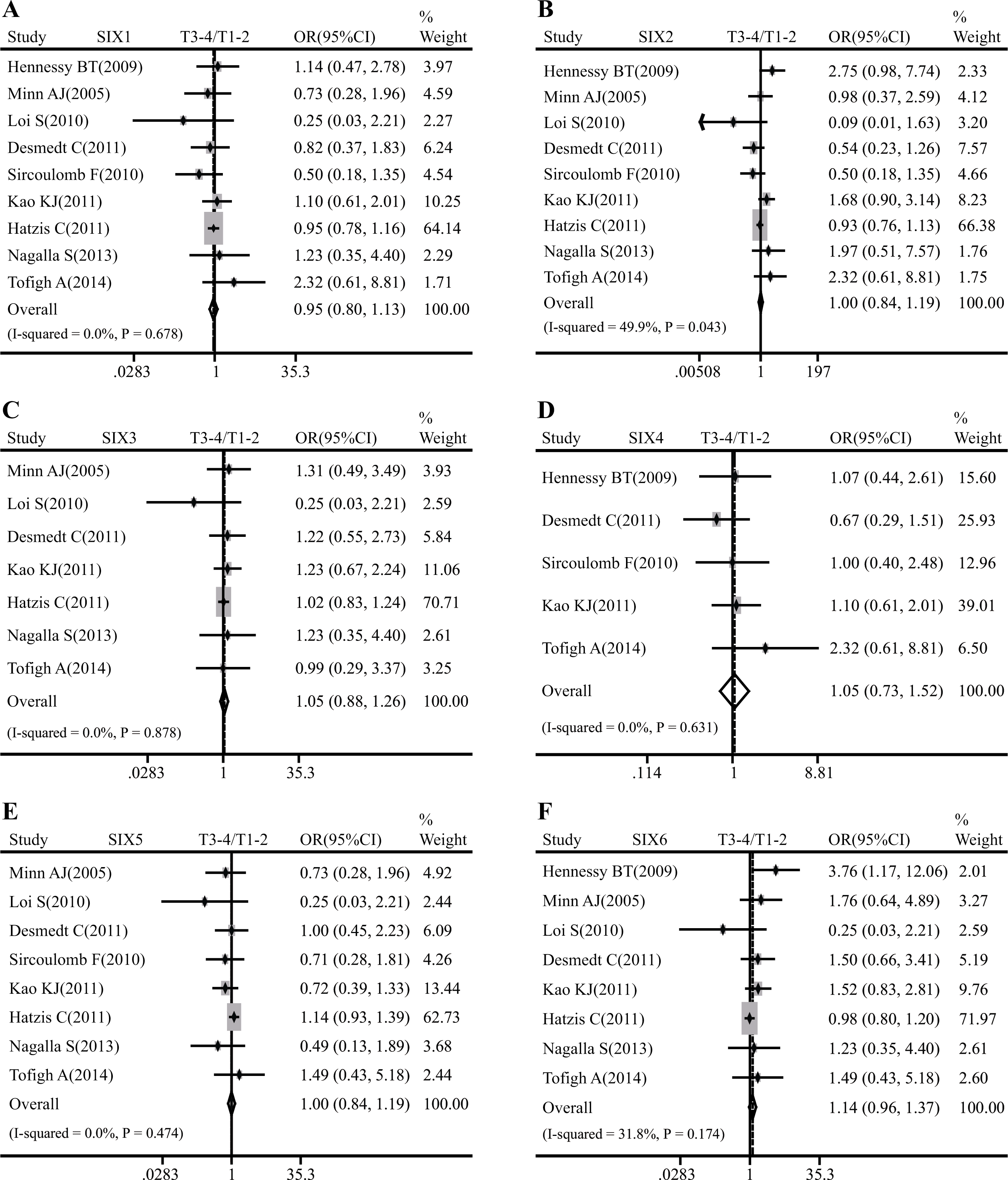


**Supplementary Figure 2** Forest plots of the odds ratio (OR) and 95% confidence interval (CI) for the association between the mRNA levels of *SIX* family members and N stage of breast cancer.


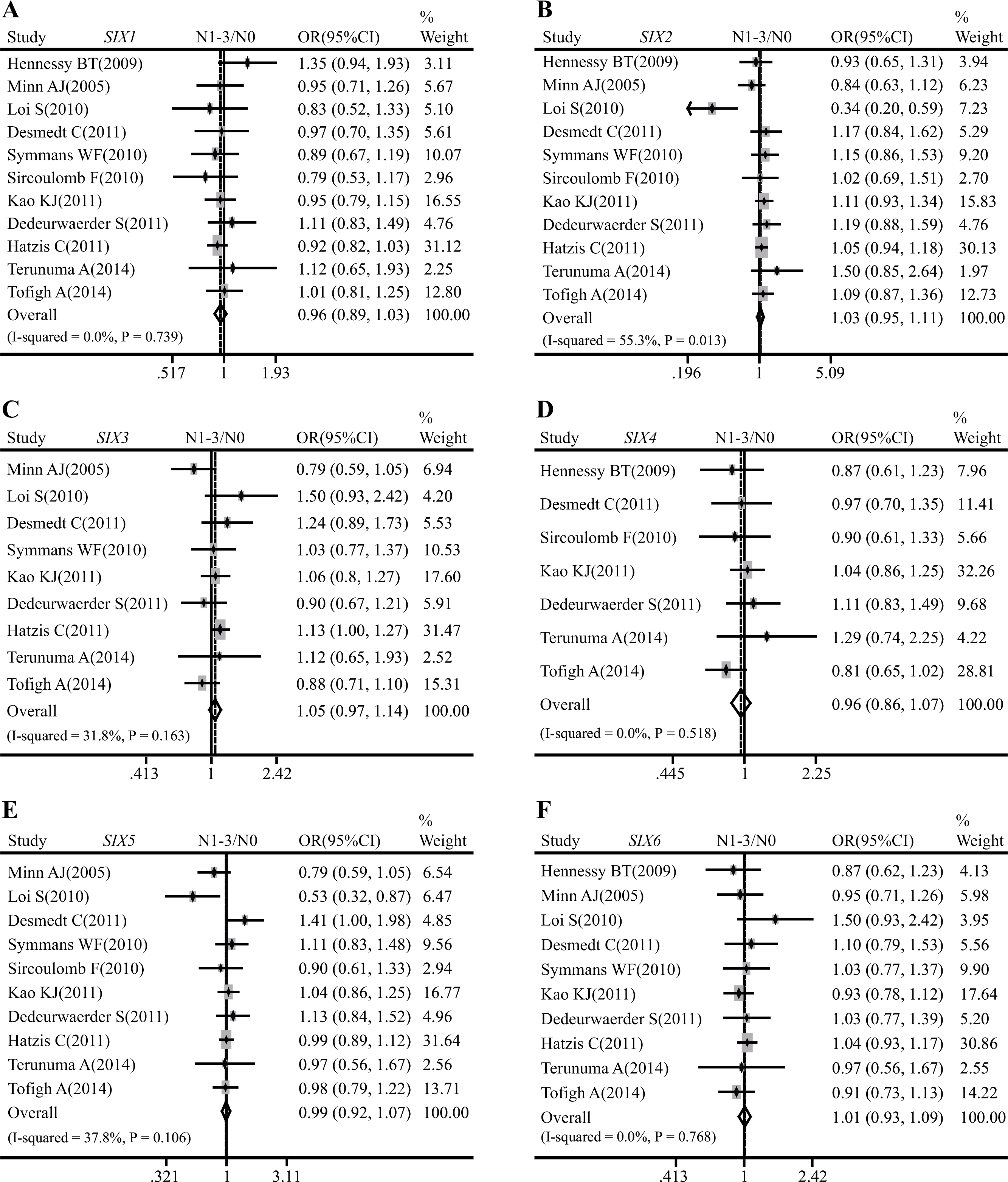


**Supplementary Figure 3** Forest plots of the odds ratio (OR) and 95% confidence interval (CI) for the association between the mRNA levels of *SIX* family members and TNM stage of breast cancer.


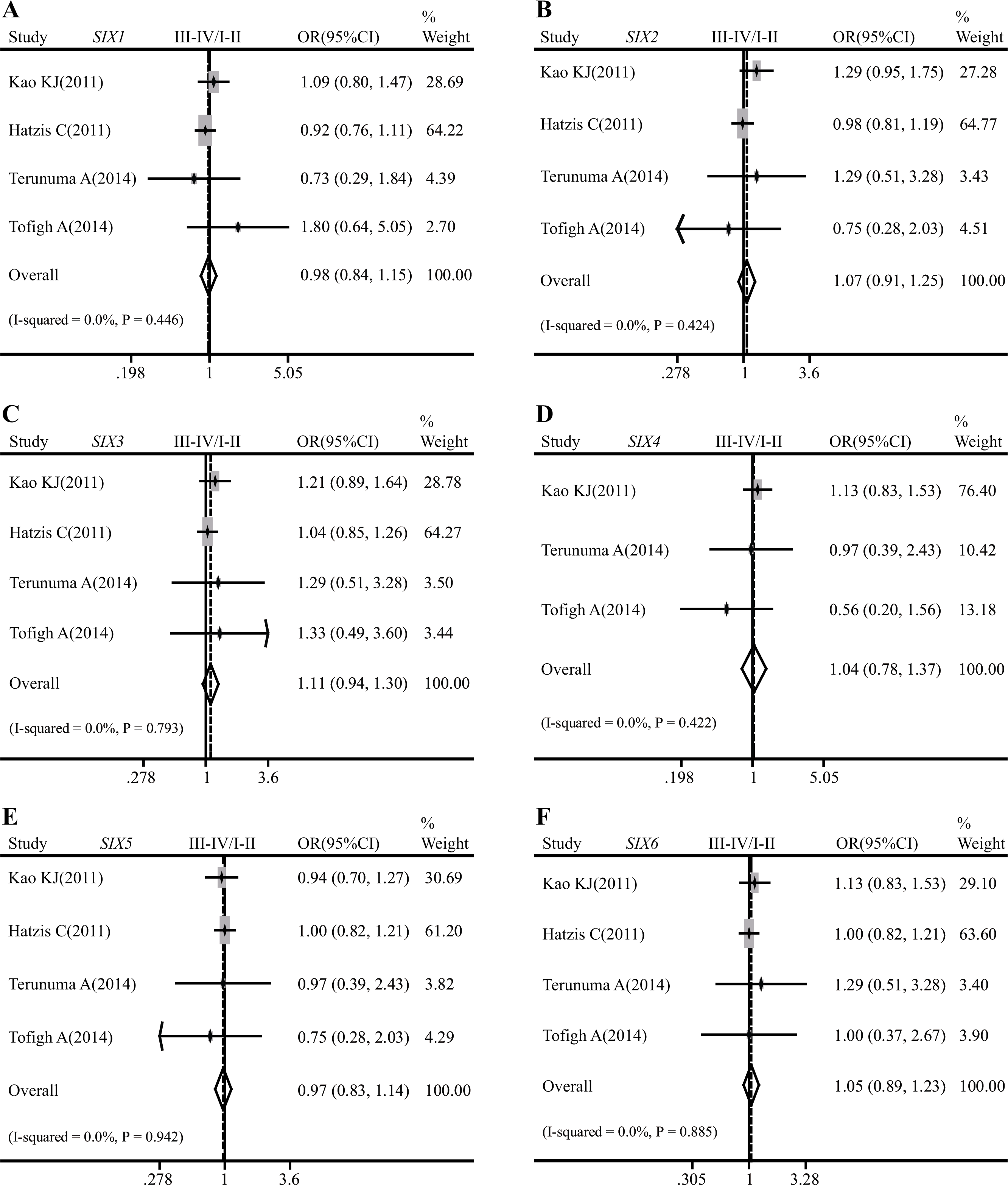


**Supplementary Figure 4** Forest plots of the odds ratio (OR) and 95% confidence interval (CI) for the association between the mRNA levels of *SIX* family members and HER2 status of breast cancer.


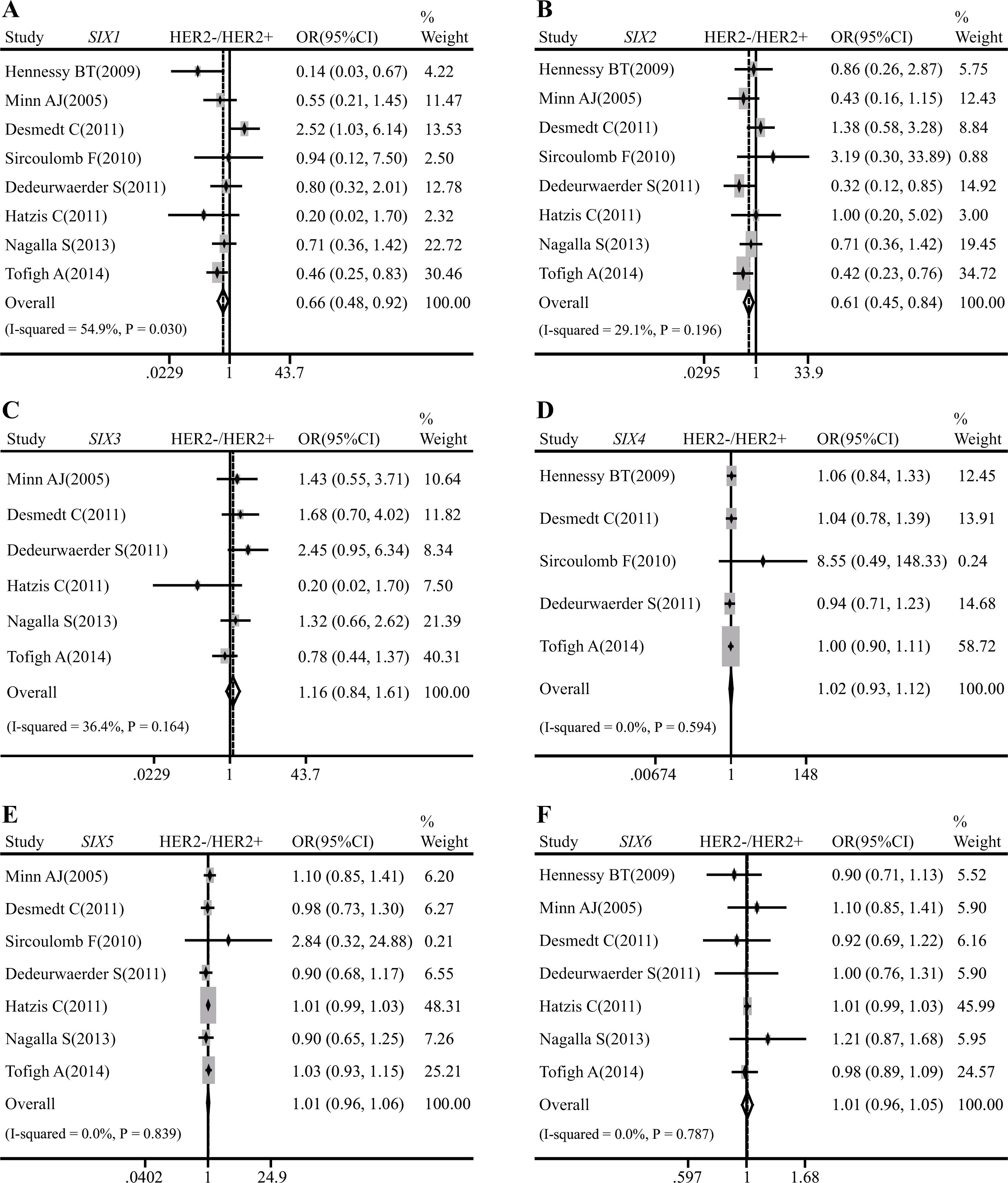


**Supplementary Figure 5** Forest plots of the hazard ratio (HR) and 95% confidence interval (CI) for the association between the mRNA level of *SIX4* and OS, RFS and MFS of breast cancer patients.


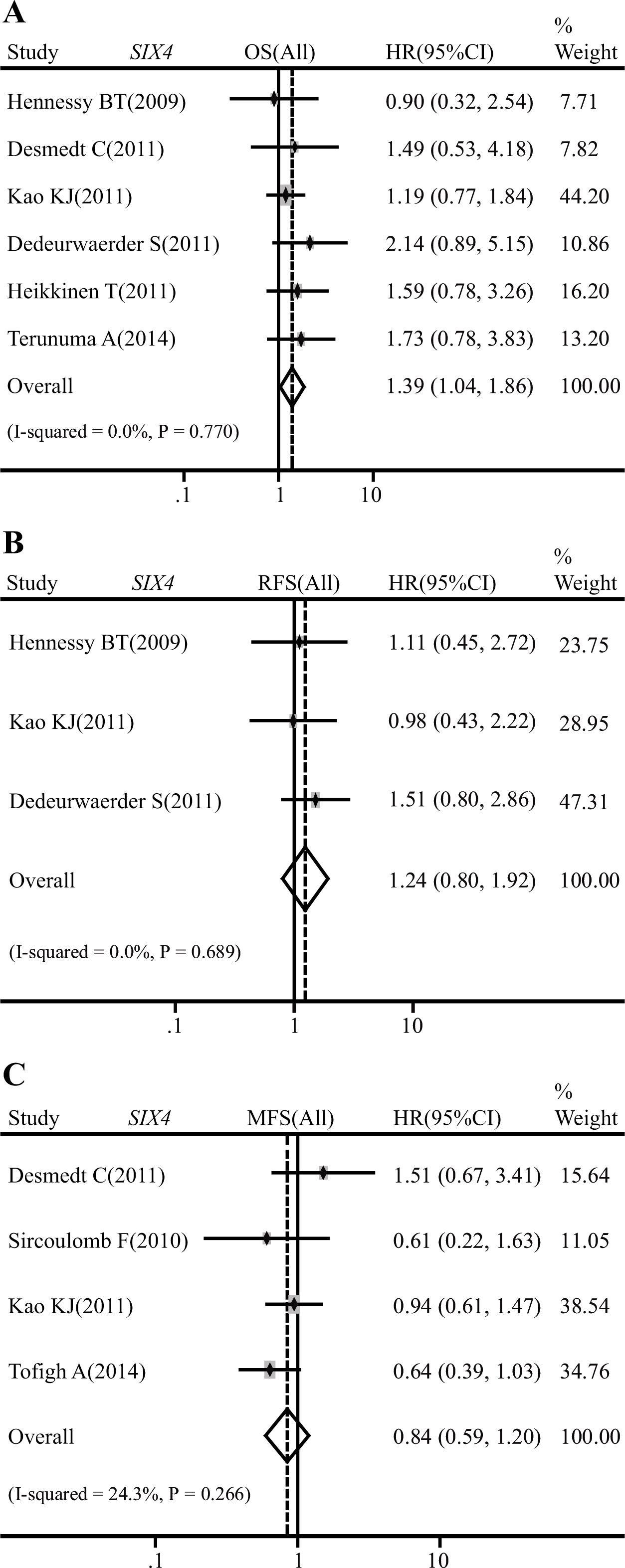

Supplement: Supplemental Digital Content [file medi-95-e4085-s001.doc]
